# Heat Induced Capsid Disassembly and DNA Release of Bacteriophage λ

**DOI:** 10.1371/journal.pone.0039793

**Published:** 2012-07-11

**Authors:** Xiangyun Qiu

**Affiliations:** Department of Physics, George Washington University, Washington, D.C., United States of America; Centre National de la Recherche Scientifique, France

## Abstract

Successive structural changes of bacteriophage 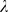 upon heating were characterized with quantitative experimental methods. In the commonly used Tris-Mg buffer, differential scanning calorimetry measurements first established that the protein capsid of 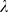 phage melts at 87°C and its genomic DNA melts at 91°C. Interestingly, prior to the capsid melting, 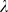
DNA was found to escape out of the capsid and subject to DNase digestion above 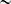
68°C, as concluded from light scattering, UV absorption, and electron microscopy studies. Further investigations indicated distinct temperature-dependent behaviors of the three phage proteins. Around 68°C, disruption of the tail first occurs and leads to the escape of 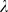 DNA; above the capsid melting temperature of 87°C, the auxiliary protein gpD of the phage head remains soluble in solution and resists centrifugal sedimentation, whereas the major capsid protein gpE is easily precipitated and likely exists as aggregates.

## Introduction

Knowledge of virus structure and assembly is of fundamental importance to decipher the life cycle of viruses and harness their virulence [1]–[4]. Viruses have also been a fertile ground to study the molecular mechanisms of macromolecular assembly owing to their “simple” structures amenable for quantitative approaches [5]–[12]. Here we examined the heat-induced disassembly of a bacterial virus, bacteriophage 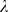. While temperature is an easily accessible parameter, there have been scarce reports on heat-induced changes of bacteriophages in the literature, e.g., only a few exist for phage HK97 and T4 [13], [14]. Characterizing the successive structural changes as a function of temperature provides insight into the principles of viral assembly, and such knowledge shall shed light on the inactivation procedure of viruses vital for food industries and pharmaceutics [15]–[17].

Bacteriophage 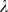 comprises of one protein capsid and one dsDNA genome (48.5 kilo-base-pair or kbp in wild-type 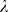 phage) [18], [19]. The protein capsid consists of an icosahedral 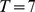 head (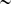
63 nm diameter) and a hollow tail (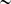
170 nm long). While 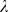 dsDNA genome is packaged in the head of the capsid, the tail of the capsid is the channel for DNA delivery when infecting bacteria [20], [21]. During the propagation of 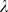 phage, the capsid head, capsid tail, and dsDNA genome are synthesized and pre-formed separately [18]. However, their assembly into a viable 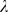 phage follows a strict sequence: 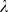 DNA is first packaged into the capsid head (also called procapsid) by its packaging motor complex and then the capsid tail is joined with the head to seal the DNA in [22], [23]. The cascades of molecular associations and dissociations in order to make a full-fledged phage are very complex and being elucidated with increasing structural and time resolution [3], [24], [25].

Disassembly of 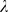 phage can be incurred by disrupting the 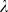 capsid or the packaged DNA, either of which suffices to inactivate the virus. Mechanically, the icosahedral capsid head is fairly stiff with a Young’s Modulus of 1.0 GPa measured by recent Atomic Force Microscopy (AFM) experiments [26]. The long tail of 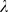 capsid is sensitive to mechanic shearing which can dislodge it from the capsid head [26], [27]. Chemically, the protein capsid is susceptible to denaturing agents such as phenol and SDS. Enclosed by the phage capsid, 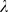 DNA is inaccessible to large molecules such as DNases, though small molecules including DNA intercalating agents can permeate through the capsid [28]. On the other hand, 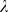 DNA is more than a passive carrier of viral genome. It is tightly packaged up to liquid crystalline density and highly pressurized [6]. The pressure of DNA increases when the salt concentrations are lowered, and this can burst open the capsid [29]. Alternatively, 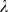
DNA can translocate through the capsid tail without damaging the capsid. This is achieved by adding the bacterial membrane receptor protein LamB to trigger the DNA ejection of 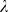 phage in vitro [30]. The initial driving force for DNA ejection is still a matter of debate with primary candidates being DNA pressure or hydrostatic pressure [6], [8]. In addition, physical approaches such as hydrostatic pressure and radiation have been experimented as a means to inactivate bacteriophages [31].

In this study, we investigated the effect of temperature on the disassembly of bacteriophage 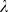 with quantitative physical and biological techniques. Working with three different phage constructs (two infectious 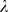 strains with different DNA lengths and one emptied 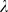 phage without DNA) in the commonly used Tris-Mg buffer [32], we determined the melting temperatures of 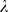 capsid and 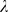 DNA to be 87 and 91°C respectively. Interestingly, 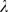 DNA appears to have escaped from the capsid around 68°C prior to the capsid melting at 87°C. We corroborated our conclusions with multiple approaches and further resolved the disruption of the phage tail as the most likely cause for the DNA escape at 68°C.

## Results

**Figure 1:**
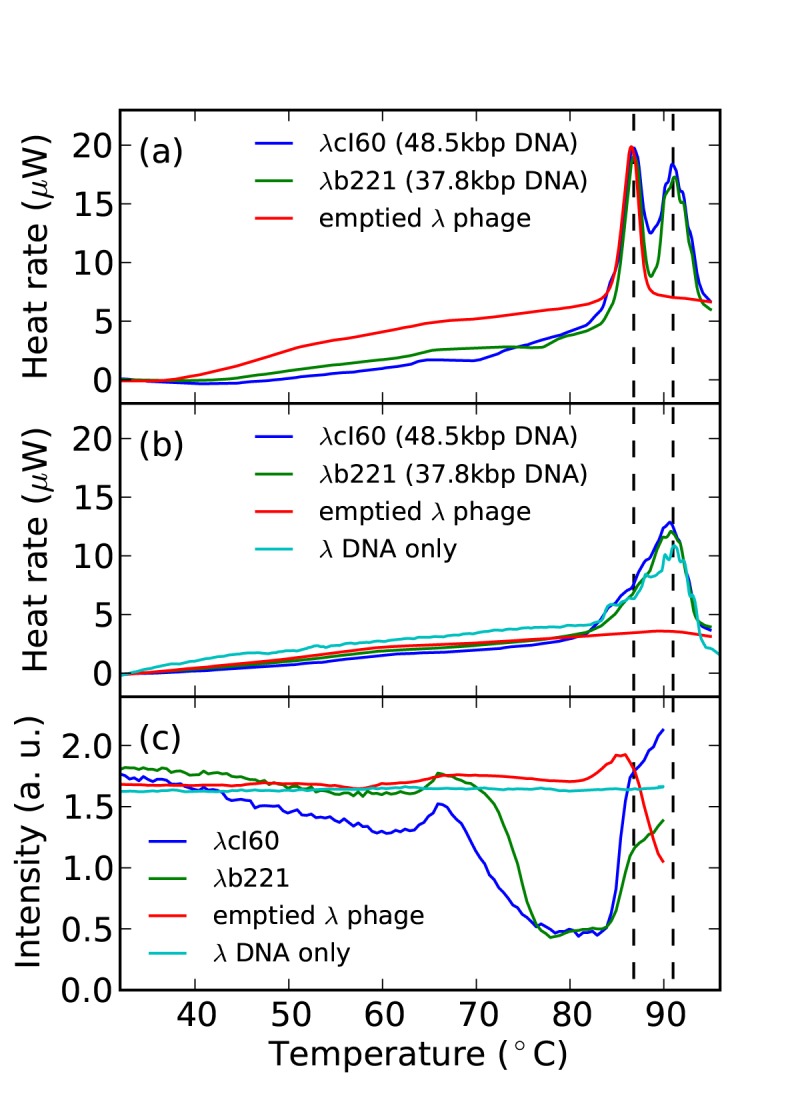
Heat induced structural transitions of *λ* phages. (a) Differential Scanning Calorimetry (DSC) data of the two intact 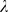 phage strains and the emptied 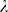 phage in the TM buffer. The two dashed vertical lines denote temperatures of 86.8 and 91°C respectively. (b) The second DSC heating scan for the same three phage solutions as in (a), with the addition of DSC data for 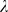 DNA only. Note the disappearance of the capsid melting peak around 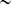
87°C. (c) Static light scattering (SLS) at the scattering angle of 90° for solutions of the 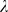 phage constructs and 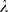 DNA only.

We first applied differential scanning calorimetry (DSC) as a thermoanalytical technique [33] to ascertain the melting transitions of 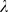 phage particles. Fig. 1a shows the heat-absorption profile of the first heating scan up to 95°C, noting that no feature was found between 95 and 125°C and thus not shown. Both intact 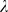 phages, 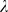 cI60 strain with 48.5 kbp DNA (100% of wild-type 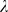 DNA length) and 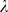 b221 strain with 37.8 kbp DNA (78%), exhibited two prominent endothermic transitions at the *same* temperatures of 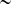
87 and 91°C. Given the known concentrations of 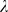 phage, we estimated that either endothermic peak accounts for 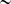
600,000 kT energy absorbed per phage particle, which is comparable to the calculated 540,000 kT per 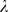 DNA based on literature data on 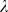 DNA melting in Sodium salt [34]. To discern the contributions from 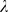 DNA and 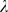 capsid, we next separately measured solutions of purified 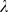 DNA or emptied 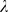 phage alone. A single melting temperature was observed for either 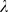 DNA (91°C, see Fig. 1b) or emptied 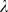 phage (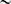
87°C, Fig. 1a). For all that, the protein capsid and the DNA genome of 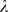 phage appear to melt independently of each other, with melting temperatures of 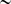
87 and 91°C respectively.

The melting of 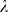 DNA observed here is consistent with the extensive studies in the literature [35]. The slightly “rugged” profile of the melting peak arises from the existence of non-random-sequenced DNA domains melting at different temperatures [36]. Remarkably, the melting of 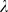 capsid occurs sharply at a single temperature (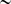
87°C), while two melting events may have been expected for the capsid. The first is the disassembly of the capsid into its protein subunits and the second is the denaturation of the protein subunits. The observation of a single melting temperature suggests the two events are coupled. Previous studies of T4 and HK97 phages also observed a single melting temperature for their respective caspids [13], [14]. Duda *et al.* in Ref. [14] discussed that the capsid subunits are stabilized by inter-subunit interactions and the disassembly of the capsid leads to concurrent denaturation of its subunits. The same is likely true for 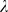 capsid, i.e., the protein subunits become unstable after capsid disassembly and melt immediately. Moreover, the capsid melting process is usually irreversible, as the capsid assembly process involves coordinated steps such as protein scaffolding, protease digestions [2]. Fig. 1b confirms that the capsid melting peak disappears during the second DSC heating scan while the 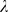 DNA melting peak persists.

Nearly identical *capsid* melting behaviors were observed from 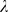 capsids containing 0%, 78%, and 100% DNA (Fig. 1a). This is somewhat surprising because the confined DNA is expected to exert stress on the capsid due to the pressurized DNA packaging, e.g., 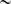
40 atm with 100% genomic DNA [37]. The packaged DNA has been recently shown to contribute considerably to the mechanical stiffness of 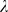 capsid, e.g., the Young’s modulus of the capsid doubles when DNA content increases from 0% to 100% [26]. Even so, we may not be able to directly compare the heat-driven melting and the mechanical elasticity because the associated molecular events may well be very different. On the other hand, the pressurized DNA packaging entails that DNA may escape if the elevated temperature induces local disruptions of the capsid (such as a lesion) prior to the capsid melting. The DNA escape prior to capsid melting would explain the independence of capsid melting on the DNA content. Additionally, the escape of DNA is not of melting nature and would not have been detected by DSC measurements.

We next applied static light scattering (SLS) as a structural probe to examine the possibility of early DNA escape. SLS is sensitive to molecular association or dissociation events, giving rise to intensity rise or drop respectively. Fig. 1c shows the SLS intensities at a fixed scattering angle for the two intact 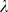 strains and the emptied phage. As expected, only one molecular dissociation event (i.e., SLS intensity drop) was observed for the emptied phage at its melting temperature of 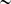
87°C. The control experiment with 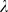 DNA alone showed no intensity change in the measurement range of 20 and 90°C (Fig. 1c). Both intact 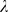 strains (cI60 and b221) exhibited an intensity drop at 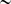
68°C, considerably below the capsid melting T of 87°C. At 68°C, neither capsid nor DNA has melted yet, the observed molecular dissociation event is consistent with the escape of DNA out of the capsid. The two intact 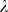 strains later display an SLS intensity rise at 87°C. We do not know its origin and attribute it to the possible association of the melted capsid and DNA since such rise was not observed in the case of emptied phages. Importantly, the SLS intensity drop at 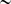
68°C supports the escape of DNA prior to the capsid melting.

The dissociation of the 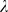 DNA and capsid prior to 87°C was further investigated by using the fact that DNA would subject to DNase digestion when outside the capsid. The amount of digested nucleotides can be quantified with UV absorption measurement after removing the 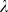 phage (emptied or full) via filtration or centrifugation. Our experimental steps are: i) each 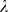 phage solution was incubated at room temperature, 72°C (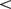
87°C), and 92°C for 10 minutes; ii) after cooling to room temperature, DNase I was added to each solution to 30 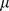
g/ml and the solution was further incubated at 32°C for 30 minutes; iii) each solution was centrifuged at 20,000×g for 90 minutes, and the supernatant was immediately measured with a UV spectrophotometer to quantify the DNA concentration via absorption at 260 nm. As expected, we found no DNA in the supernatant of the phage solution incubated at room temperature, i.e., no DNA escaped and all phages sedimented. On the other hand, 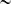
80% of the total 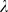 DNA appeared in the supernatant from 72°C incubation, and nearly all 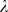 DNA was found in the supernatant from 92°C incubation. This corroborates the dissociation of 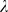 DNA from the capsid before (and after) the melting of the capsid at 87°C.

Now it is established that 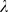 genomic DNA escapes 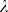 capsid prior to the capsid melting, however, little is known about the mechanisms, i.e., how does 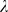 DNA escape the not-yet-melted capsid? We then applied Electron Microscopy (EM) to examine the structures of the two intact 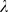 phages (cI60 and b221) at three temperatures (room temperature, 72, and 92°C) without the addition of DNase I. The EM results are not shown here because they do not provide qualitatively new information. Nonetheless, our EM study first confirmed the escape of DNA above 72°C by indicating the appearance of gel-like matrix likely made up of 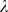 DNA. Moreover, an intriguing observation, though far from being conclusive, is the possible disruption of the capsid tail above 72°C. Dislocation of the capsid tail from the head, if it occurs, makes it possible to separate the two components via centrifugation because of their different sedimentation properties. For example, the phage tail was measured to give 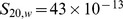 second, while the phage head without DNA has 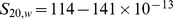 second [38], [39]. Protein gel electrophoresis can then be used to identify the separated components, owing to the “simple” composition of 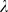 capsid with only three types of large copy-number proteins [18], [40]. Two of the three are in the capsid head: gpD (110 amino acids, 405 copies) and gpE (341 amino acids, 405 copies). One is in the tail: gpV (246 amino acids, 192 copies). We followed an experimental protocol similar to the UV absorption assay discussed earlier. In brief, 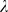 phage solutions were incubated separately at room temperature, 72, or 92°C for 10 minutes. Each solution was then incubated at 32°C for 30 minutes with DNase I. Then, 1/3 of the total volume is set aside as the “as-is” fraction, and the rest 2/3 was centrifuged at 20,000×g for 90 minutes at 4°C, which was chosen so that empty 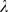 phage heads near the top (if present) would travel 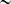
8 mm to reach the tube bottom. The top half was quickly transferred to another tube as the “supernatant” fraction, and the bottom half was kept as the “pellet” fraction.

**Figure 2:**
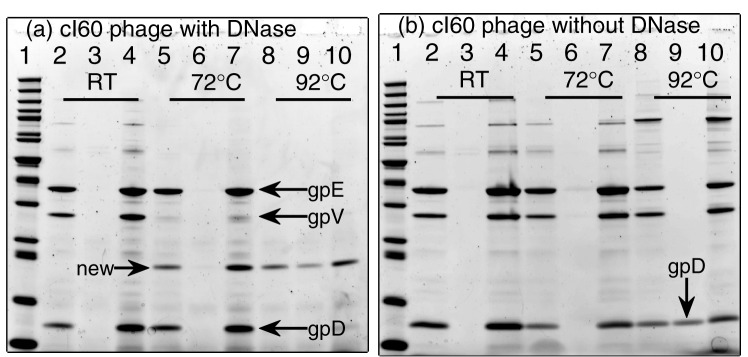
SDS-PAGE of intact cI60 phages incubated at room temperature (RT), 72, and 92°C. (a) With addition of DNase I. (b) Without the addition of DNase I. The three fractions incubated at each temperature are shown in the order of the as-is, supernatant, and pellet fractions (see text for details). NuPAGE 12% Bis-Tris gels (Invitrogen) were run at 120 V for 2 hours and 45 minutes in the MOPS SDS buffer. Lane #1 is the PageRuler unstained protein ladder with 10 kDa to 200 kDa range.

Fig. 2a shows the sodium dodecyl sulfate polyacrylamide gel electrophoresis (SDS-PAGE) of the three fractions (“as-is”, “supernatant”, and “pellet”) of the 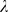 cI60 strain. The room temperature fractions are in lanes #2–4. Lane #2 is factually the intact cI60 phage stock. Its three pronounced bands correspond well to the gpD, gpV, and gpE proteins as graduated by the protein ladder in lane #1. The supernatant fraction (lane #3) is void of any protein, confirming that centrifugation effectively sediments all phage particles. The pellet fraction (lane #4) is essentially a two-fold concentrated solution of the “as-is” fraction (lane #2). Accordingly, lane #4 shows bands at the same locations, but brighter than in lane #2. Lanes #5–7 show the three fractions after incubation at 72°C. Compared with the room temperature fractions, the head proteins gpD and gpE exhibited the same patterns, consistent with the still intact capsid heads at 72°C. However, the tail protein gpV disappeared in all three fractions (lanes #5–7), and a new protein band (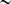
170 amino acids) appeared in all but the supernatant fractions. When incubated at 92°C (lanes #8–10), all three proteins (gpD, gpV, and gpE) disappear in all fractions, and the same new protein as observed at 72°C now exists in all fractions. The melting of head proteins (gpD and gpE) does not appear to contribute to the new protein band because the bend intensity is comparable to that at 72°C. The new protein band is presumably a breakdown product of gpV protein, though its absence in the supernatant fraction (lane #6) suggests its association with itself or other molecules.

**Figure 3:**
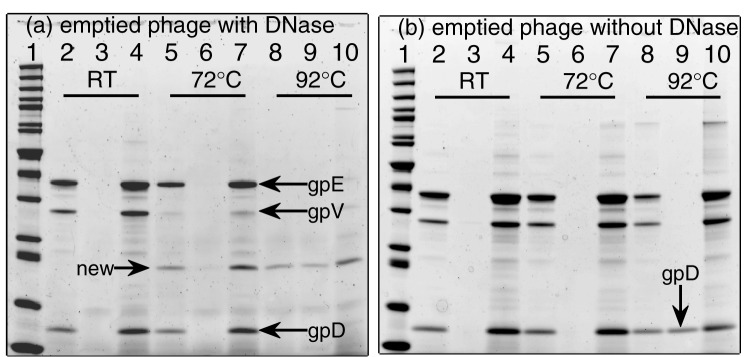
SDS-PAGE of emptied 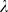 phages incubated at room temperature (RT), 72, and 92°C. (a) With addition of DNase I. (b) Without the addition of DNase I. Gel conditions and lane annotations are as in Fig. 2.

While the different behaviors of the capsid proteins are interesting, their partial breakdown or complete disappearance were puzzling. It was brought to our attention that many DNase I preparations may carry non-negligible amount of protease compounds. We then performed the same experiments without DNase I and show the results in Fig. 2b. Now all three proteins are present at all temperatures, confirming the residue protease activity of DNase I solutions. Comparisons of the results in Fig. 2a&b provide additional information on the temperature-driven disassembly of phage 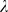. The tail protein gpV appears to the first one to be disrupted and subject to protease digestion at 72°C. The capsid head proteins (gpE and gpD) are susceptible to protease digestion at 92°C after the cooperative melting at 87°C determined from DSC data. Without protease digestion, the gpD protein appears to largely soluble and resist centrifugal sedimentation (lane #9 of Fig. 2b). The gpE protein completely goes to the “pellet” fraction and likely exists as aggregates. In addition, we repeated the same SDS-PAGE experiments for the emptied 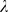 phage, and the same behaviors were observed (Fig. 3a&b). This establishes the early disruption of the capsid tail below 72°C as a process independent of its DNA content.

## Discussion

Interrogating temperature-driven structural transitions of bacteriophage 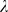 with collective methods and distinctive phage constructs, we found that, upon heating, the capsid tail is first disrupted around 68°C which presumably triggers the release the 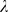 DNA; the head of the capsid melts later at 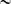
87°C, exhibiting a strong endothermic peak; at 91°C, 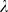 DNA melts. The melting temperature of 87°C for 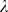 capsid is comparable with that of other characterized viral capsids, for example, 89°C for T4 [13], 93°C for HK97 [14]. Importantly, we consistently observed that the melting of 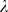 capsid head is independent of the amount of packaged DNA inside the capsid (e.g., essentially no difference between 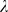 capsids with 0%, 78%, or 100% DNA length). This is not surprising as we now know that 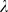 DNA escapes around 68°C, prior to the melting of the capsid head. The insensitivity of capsid melting to DNA content has also been reported for phage HK97, where Duda *et al.* stated that “HK97 heads release their DNA at temperatures well below the onset of DNA melting or capsid denaturation” [14]. One difference is that the intact capsid tail was suggested as the route of DNA release for HK97 phage [14]. In the case of 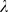 phage, the susceptibility of tail protein gpV to protease digestion above 68°C suggests substantial structural changes and very likely dislodging of the phage tail prior to the DNA release.

In sum, our study provides the first quantitative analysis of the melting of bacteriophage 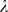 upon heating, and contributes to the scarce literature on thermal-driven structural transitions of bacteriophages. Characterization of the molecular events of different nature would not have been possible without combining complementary techniques (e.g., thermoanalytical and structural). We think that the early escape of DNA genome prior to the melting of viral capsids may be a general feature of the melting of dsDNA bacteriophages in which DNA-capsid interactions are weak [27]. One novelty of the 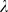 capsid is its two-step structural transitions at 68 and 87°C. This also reveals the fragility of the capsid tail, and the relatively moderate temperature of 68°C may suggest a convenient method to eradicate 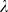 phage in various applications.

## Materials and Methods

### Preparation of Intact 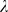 Bacteriophage and Emptied Phage

As described previously [30], Luria-Bertani (LB) cultures of E. coli strain c600 were infected with 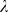 phage stock in the early exponential growth phase (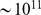 cell/liter), with a multiplicity of infection of 0.1. Cell density first increased and then dropped due to bacterial lysis for phage release; chloroform was added before harvest to lyse the remaining cells; and cell debris was removed by gentle centrifugation. Phage particles were precipitated by 10% poly-ethylene-glycol (PEG) 8000 and purified by CsCl equilibrium density gradient. The CsCl salt was then substituted with the TM buffer (50 mM Tris pH 7.5, 10 mM MgCl_2_) by equilibrium dialysis at 4°C with four buffer changes over one week.

Emptied 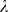 phage was prepared by in vitro DNA ejection of infectious 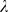 phage in the TM buffer [30]. Phage stock was mixed with bacterial membrane receptor protein LamB and then incubated at 32°C over night. Small amount of DNase I and 1% detergent (oPOE) were added to digest the ejected DNA and disperse the LamB protein respectively. The reaction mixture was purified by CsCl equilibrium density gradient, and the emptied phage fraction was dialyzed extensively against the TM buffer to make emptied 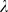 phage stock.

### Differential Scanning Calorimetry (DSC)

DSC measurements were carried out with the N-DSC III calorimeter by Calorimetry Science Corporation. Intact phage or emptied phage solutions (800 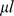, 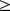
3
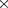
10^12^ particle/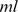) were degassed before loading (cell volume 630 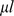). Temperature scan rate was 0.5°C/min for both the heating and cooling cycles between 10 and 95°C. Heating up to 125°C was performed for selected samples to verify the absence of transitions above 95°C.

### Static Light Scattering (SLS)

SLS intensities of phage solutions as a function of temperature were measured with the Fluoromax-3 fluorimeter by Jobin Yvon by setting emission and excitation wavelengths to be the same (360 or 600 nm). Temperature was scanned from 20 to 90°C as allowed by its Peltier temperature controller. Scattering at 90° angle was collected at every 0.5°C with the exposure time of 0.5 second.
